# Engaging family caregivers and health system partners in exploring how multi-level contexts in primary care practices affect case management functions and outcomes of patients and family caregivers at end of life: a realist synthesis

**DOI:** 10.1186/s12904-021-00781-8

**Published:** 2021-07-16

**Authors:** Grace Warner, Lisa Garland Baird, Brendan McCormack, Robin Urquhart, Beverley Lawson, Cheryl Tschupruk, Erin Christian, Lori Weeks, Kothai Kumanan, Tara Sampalli

**Affiliations:** 1 School of Occupational Therapy, Dalhousie University, P.O. Box 15000, Halifax, Nova Scotia B3H 4R2 Canada; 2 Faculty of Nursing, University of Prince Edward Island, 550 University Avenue, Charlottetown, PEI C1A 4P3 Canada; 3 School of Health Sciences, Queen Margaret University, Queen Margaret University Drive, Musselburgh, EH21 6UU Scotland; 4 Department of Community Health and Epidemiology, Dalhousie University, 5790 University Avenue, Halifax, NS B3H 1V7 Canada; 5 Department of Family Medicine, Dalhousie University, 1465 Brenton Street, Suite 402, Halifax, Nova Scotia B3J 3T4 Canada; 6 Palliative Care Integration, Nova Scotia Health Authority, 530C Bethune Building, 1276 South Park st, Halifax, NS Canada; 7 Primary Health Care Implementation, Nova Scotia Health Authority, 6960 Mumford Road, Suite 2068, Halifax, NS B3L 4P1 Canada; 8 School of Nursing, Dalhousie University, P.O. Box 15000, Halifax, Nova Scotia B3H 4R2 Canada; 9 Palliative Care Integration, Nova Scotia Health Authority, Room 522 Bethune Building, 1276 South Park St, Halifax, NS B3H 2Y9 Canada; 10 Research, Innovation and Discovery, Nova Scotia Health Authority, Halifax, Canada

**Keywords:** Realist synthesis, Primary care, Primary palliative care, Case management, Program theories, End-of-life communication, Advance care planning, Health system partners, Family advisors, Family caregivers

## Abstract

**Background:**

An upstream approach to palliative care in the last 12 months of life delivered by primary care practices is often referred to as Primary Palliative Care (PPC). Implementing case management functions can support delivery of PPC and help patients and their families navigate health, social and fiscal environments that become more complex at end-of-life. A realist synthesis was conducted to understand how multi-level contexts affect case management functions related to initiating end-of-life conversations, assessing patient and caregiver needs, and patient/family centred planning in primary care practices to improve outcomes. The synthesis also explored how these functions aligned with critical community resources identified by patients/families dealing with end-of-life.

**Methods:**

A realist synthesis is theory driven and iterative, involving the investigation of proposed program theories of how particular contexts catalyze mechanisms (program resources and individual reactions to resources) to generate improved outcomes. To assess whether program theories were supported and plausible, two librarian-assisted and several researcher-initiated purposive searches of the literature were conducted, then extracted data were analyzed and synthesized. To assess relevancy, health system partners and family advisors informed the review process.

**Results:**

Twenty-eight articles were identified as being relevant and evidence was consolidated into two final program theories: 1) Making end-of-life discussions comfortable, and 2) Creating plans that reflect needs and values. Theories were explored in depth to assess the effect of multi-level contexts on primary care practices implementing tools or frameworks, strategies for improving end-of-life communications, or facilitators that could improve advance care planning by primary care practitioners.

**Conclusions:**

Primary care practitioners’ use of tools to assess patients/families’ needs facilitated discussions and planning for end-of-life issues without specifically discussing death. Also, receiving training on how to better communicate increased practitioner confidence for initiating end-of-life discussions. Practitioner attitudes toward death and prior education or training in end-of-life care affected their ability to initiate end-of-life conversations and plan with patients/families. Recognizing and seizing opportunities when patients are aware of the need to plan for their end-of-life care, such as in contexts when patients experience transitions can increase readiness for end-of-life discussions and planning. Ultimately conversations and planning can improve patients/families’ outcomes.

**Supplementary Information:**

The online version contains supplementary material available at 10.1186/s12904-021-00781-8.

## Background

For life-limiting conditions, such as end stage chronic illnesses, the ability to predict the prognosis is often challenging [1, 2]. To address this uncertainty and to provide higher continuity of care that is in alignment with patient values and preferences, initiating an upstream approach to palliative care in the last 12 months of life has been recommended [3]. This approach blends palliative care and chronic disease management approaches [3–6]. Implementing palliative care earlier can improve patient quality of life, symptom management, depression, and in some cases increase life expectancy [7–9]. Primary care practices, as the first point of contact to the health system, provide comprehensive primary health care to their patients, from birth to end of life (https://accreditation.ca/standards/). As part of a comprehensive approach, primary care providers support their patients at end-of-life through primary palliative care (PPC) [10]. Essential elements of PPC often include patient and family-centred communication; patient and family education about illness trajectory and prognosis; goals of care and advance care planning; psychosocial and spiritual support; and end-of-life care [10, 11]. PPC is part of a palliative approach to care [4] and can assist patients and their families who are struggling to manage the high symptom burden, often due to multi-morbidities and increasing frailty that often occurs at end-of life.

Case management functions can support the delivery of PPC and help patients and their families navigate health, social and fiscal environments that become more complex at end-of-life. They can also help the integration of care across organizations and within teams, improving continuity of care [12]. According to the National Case Management Network (2009), case management is a client-driven, collaborative process that ensures effective and efficient use of resources for the provision of quality health and social support services. The Canadian Standards of Practice for Case Management have detailed both the competencies and functions of case management; the competencies include case management expert, communicator, collaborator, navigator, manager, advocate and professional; case management functions include client identification and eligibility for case management services, assessment, planning, implementation, evaluation, and transition [13]. Implementing case management functions facilitate the ability of inter-professional teams to work collaboratively and in partnership with clients and their families to identify goals of care [13–15].

Applying case management functions to deliver PPC has potential to assist providers with identifying patients and their families who need a palliative approach to care, assessing patient and family needs, and developing a plan to address the identified needs. This aligns with PPC elements of improving communication, providing education, and developing a comprehensive advance care plan that helps patients and families manage life-limiting chronic diseases, by increasing their knowledge of the complexities of their conditions and providing needed skills for accessing available services and supports [16].

A comprehensive advance care plan can be a care plan for end-of-life that allows patients to share their personal values, goals, and preferences for medical care, ensuring the three are in alignment [17–19]. Having a comprehensive advance care plan facilitates case management functions that can increase referrals to community-based resources and access to care from inter-disciplinary providers, while also improving the timeliness and cost-effectiveness from accessing appropriate resources [14]. Having an advance care plan in place has been found to reduce unnecessary admissions to hospitals and emergency departments [20], and improve the possibility of a home death by increasing the ability of families to cope [21]. Implementing this plan early in primary care can further increase the likelihood that patients’ goals of care can be met [22, 23].

A realist synthesis was conducted to understand how contexts affect the implementation of case management functions in primary care to improve the delivery of PPC and end-of life patient and family/friend caregiver outcomes. A realist synthesis considers programs as theories, because programs are implemented on a hypothesis of ‘if we do X in this way, then it will bring about outcome Y’ [24, 25]. It involves both searching through the literature and drawing on experience from non-academic partners to identify concepts and develop theories that provide some explanation about how the program of interest may work. Ultimately, a realist synthesis is concerned with uncovering ‘what works’ within differing contextual configurations [26].

The goal for this realist synthesis was to synthesize evidence on how multi-level contexts and mechanisms affected the implementation of the case management functions of patient identification, assessment, and planning, as they relate to initiating end-of-life conversations, assessing patient and caregiver needs, and patient/family centred planning in primary care practices to improve outcomes. In addition, the synthesis explored how these functions aligned with critical community resources. For the purpose of our study, we identify critical community resources as community-based resources that palliative patients and their families identified as most important for end-of-life care.

## Methods

### Research team

The full research team included academic researchers who had expertise in family medicine, occupational therapy, nursing, case management, palliative approach to care, volunteer palliative care services, realist review methodology, and health system change. The two research leads (GW, LGB) were involved in all processes of the review and incorported strategies to ensure study rigor from the outset. These included investigator responsiveness, methodological coherence, and an active analytic stance [27]. The research leads ensured responsiveness and maintaining an active analytic stance by remaining open, using sensitivity, creativity and insight to support them to relinquish any ideas that were poorly supported by the review data. This was achieved through lead researchers’ weekly reflective discussions and documentation of analytic and review process documentation throughout the research process. Methodological coherence was achieved by following Realist and Meta-narrative Evidence Syntheses—Evolving Standards (RAMSES) [28] realist review standards and consultation with our academic research partners with realist review expertise.

RAMESES training materials acknowledge the need to involve the ‘commissioner’ or knowledge user of the review in all aspects of identifying the research question, literature search, and developing recommendations based on the findings. Our non-academic research partners included health system administrative leads for primary health care (EC, TS) and palliative care (CT) (*n* = 3), and family advisors (*n* = 2) who had experiences with family members facing end-of-life. Our non-academic partners were not study participants as they were not the focus of the research or collection of data. Rather they were an integral part of the research team who were engaged and consulted at every stage of the review, and their input was woven into our research process. As the role of our non-academic research partners in this review was consultative in nature, a research ethics board review of our study was not required or sought.

As outlined in the quality standards for realist reviews [28] stakeholder involvement is an important step when focusing the scale of the review, as it maximizes the relevance to the end-user. The goal and scope of the review were created and refined through consultations with our non-academic research partners. The health system administrative leads helped finalize search terms and educated academic team members on how findings from the review could inform health system initiatives and policies related to PPC. The family advisors were asked to share personal experiences of caring for a family member at end-of-life and reflect on how these experiences could inform review processes and findings. They were also asked to help us define what were “critical community resources”. Consultations with non-academic research partners occurred throughout the review using one-to-one conversations, email, and team meetings. Quarterly team meetings lasted from one to 2 hours; they were used to formally present and discuss evolving program theories. Feedback was documented in meeting minutes and used to guide next steps of the review.

### Realist review process

Realist reviews are theory driven. The process is iterative and fluid where program theories are created and refined to hypothesize how, why, and in what particular contexts programs work to improve outcomes. This process of refinement is achieved by proposing possible program theories, searching the literature to find evidence to test those theories, analyzing and synthesizing the evidence, assessing whether program theories are supported and plausible, and making revisions as necessary.

Pawson expresses program theories as C-M-O configurations of how particular contexts (C) catalyze mechanisms (M) to generate improved outcomes (O) [25]. Dalkin’s (2015) work explains that mechanisms are related to both program resources M (Resources) that are introduced into a specific context (C) and to individual reasoning or reactions M (Reasoning) triggered based on the interaction between M (Resources) and Context [29]. Depending on whether the interaction is enabling or disabling determines whether the Outcome is positive or negative. Incorporating Dalkin’s [29] work into the original C-M-O configuration identified by Pawson [25], leads the following configuration: M (Resources) + C interaction leads to M (Reasoning) = O. This configuration was used in the review when identifying reoccurring patterns or demi-regularities in the literature to create program theories.

Our realist review is based on Pawson’s five iterative stages: (1) locating existing theories, (2) searching for evidence, (3) selecting articles, (4) extracting and organising data and (5) synthesising the evidence and drawing conclusions [30]. To ensure our review processes were consistent with a realist approach, the team followed the methodology articulated in the Realist and Meta-narrative Evidence Syntheses—Evolving Standards (RAMESES) training documents [31], and consulted with team members (RU, BM) who were experts on realist syntheses. Finally, the Quality Standards for Realist Synthesis form [31] was completed and discussed within the team during program theory development. Consecutive cycles of searching, locating, extracting, and evaluating research and grey literature were conducted to determine if the evidence supported or refuted program theories. Pawson’s five iterative stages have been used to organize the methods section.

### Step 1: locating existing theories

Initial program theories were created at the beginning of the review process in a brainstorming meeting with our non-academic research partners. The meeting identified initial theories to understand how the mechanism of case management connects patients and their family/friend caregivers early in their trajectory toward end of life to health services and critical community resources to improve their outcomes. These theories were also informed by the Canadian Standards of Practice for Case Management literature [13, 19, 32] on case management functions and competencies. Key case management competencies that aligned with our theories were communication, collaboration, and navigation. Case management functions were considered separately, rather than as the responsibilities of one individual who would act in the role of case manager. Our non-academic research partners and other stakeholders were engaged with refining our outcomes priorities and relevant contexts for our program theories.

#### Refining outcome priorities

The initial program theory outcomes needed to be refined to narrow the focus of our review. To achieve this objective and ensure our outcomes were relevant to our end-users a modified Delphi process was conducted. The modified Delphi process involved our non-academic research partners, in addition a purposive sample of seven other stakeholders who represented health system administrators, primary and palliative care practitioners, and community organizations focused on palliative care were involved. The following steps were followed. First the entire research team created a list of relevant outcomes that was clustered into categories and sub-categories to improve readability. Secondarily the list was shared with participants who prioritized outcomes and provided reasons for their prioritization. Finally, research leads reviewed responses and compiled a final list of outcomes that were linked to improved patient quality of care, caregiver bereavement, and represented all stakeholders’ perspectives. The final outcomes were improved patient and caregiver involvement in plan of care, policy frameworks that supported integrated end-of-life care, health practitioners from different sectors sharing the responsibility of case management functions and coordination of care, and the implementation of evidence-based palliative approaches to care. These outcomes were subsequently used to categorize outcomes during data extraction and to test program theories for relevancy.

#### Identifying relevant contexts

To guarantee all relevant contexts (C) would be considered in our program theories, Pawson’s work was reviewed to generate a list of context categories that might apply to the review [25, 30]. Contexts were grouped into individual capacities (i.e., practitioner capacities), interpersonal relationships (i.e., primary care team relationships), institutional setting (i.e., primary care setting), and infra-structure (i.e., health system policies or strategies surrounding primary care settings).

A video-taped brainstorming session between research team members clarified how these contexts might interact with M (Resources) to affect M (Reasoning) and generate positive or negative (O) Outcomes. The brainstorming session resulted in a conceptual diagram (see Additional File 1), the video of the session was shared with any research team members unable to attend the session. This sharing included our non-academic research partners who were asked to share the video with other stakeholders to elicit feedback on the process and resulting hypotheses. The feedback helped identify pertinent contextual factors within the health system where a readiness for change may exist. The health system already had an integrated palliative care strategy, had identified an increased readiness to implementing a palliative approach to care in primary care practices, and was already implementing palliative care education (Pallium Canada’s LEAP curriculum https://www.pallium.ca/course/leap-core/) within primary care practices. Given the health system already had an integrated palliative care strategy our review became less focused on identifying factors within the infra-structure context.

### Step 2: searching the literature

The purpose of this step was to identify a relevant body of literature for developing and refining our program theories. The Initial program theories developed in step one were explored in a librarian-assisted systematic search (see Additional File 2: A. Search terms for Initial systematic search strategy) to identify evidence supporting the theories. When initial program theories needed to be refined a second librarian-assisted systematic search (see Additional File 2: B. Second systematic search strategy for identification of studies on key topics) was conducted. At this point additional topics relevant to our review surfaced as needing further exploration to clarify concepts relevant to our program theories. To address this gap several smaller purposive searches were conducted by the research team in consultation with the librarian (see Additional File 2: C. Broad topics for librarian-assisted purposive search) and content experts. These searches explored topics in-depth that related to using case management functions to support the delivery of PPC, they included advance care planning, preferences for care, patient/family navigation, integrated/interprofessional care, shared decision making at end-of-life, end-of-life conversations, compassionate communities, and palliative approach to care.

Results from these purposive searches furthered program theory development by first identifying a definition of advance care planning that aligned with one of the community resources prioritized by family advisors. This definition incorporated the role of family, and the need to identify patient and family goals of care to inform future planning [33]. Secondarily, the searches identified literature on Health Promoting Palliative Care [34], Compassionate Communities [35], and the New Essentials model [36]. These initiatives informed program theories of how primary care services could be integrated with community resources and advance care plans created in primary care could be shared with community sectors. Literature searches were combined with citation searches on included articles.

The evidence accrued through these searches was integrated into the successive cycles of program theory refinement described in the manuscript. The quality standards discuss the balance between the search process being comprehensive versus theoretical saturation [28]. The two systematic searches provided a comprehensive search of the literature, whereas the purposive and citation searches provided more targeted searches to achieve theoretical saturation. The purposive searching of the literature is an important process in realist reviews to find additional data to confirm, refine or refute aspects of the program theories.

Records identified in the searches were entered into RefWorks reference management software (https://refworks.proquest.com) then transferred to Covidence systematic review software (https://www.covidence.org) to screen and identify included articles. All articles were screened independently by two reviewers. A two-stage process was implemented of first reviewing the title and abstract then screening the full article using established inclusion/exclusion criteria (See Table 1). Team discussions resolved any conflicts.

**Table 1:** Inclusion/exclusion criteria

**Inclusion/exclusion criteria used to identify relevant sources**	
**Inclusion Criteria:**	
English, 2009 + (chosen because year when National Case Management Network published Canadian Standards for Practice for Case Management http://www.ncmn.ca/)	
Included articles that discussed any of the following topics:	
• 1) The concept of end of life/palliative/self-management for those with high symptom burden:	
• 2) Palliative approach-early access, early identification, how to identify individuals appropriate for a palliative approach, such as tool or clinical criteria, surprise question;	
• 3) How to help transitions from chronic condition management into early palliative care or from early palliative care to higher levels of palliative care;	
• 4) Using, Identifying, or informing how to identify, critical community supports in the last year of life, e.g., may look at patient family needs and preferences;	
• 5) Interdisciplinary collaboration for early palliative care; or	
• 6) Case management or coordination of care across the continuum of palliative care delivery and settings.	
• **Exclusion criteria:** articles involving children or young adults	

### Step 3: selecting articles

All included articles records were downloaded into Excel where further organization and exploration of the articles was conducted. The realist review quality standards stipulate the importance of relevance and rigor. Relevance is defined as whether the literature can contribute to program theory building or testing. Rigor is whether the methods used to generate the data are credible and trustworthy. Rigor includes the assessment of the quality of the studies that is more critical to systematic reviews. The Excel spreadsheet captured information on the study design used to assess the level or quality of evidence as categorized by Tomlin and Borgetto (2011) [37], and on its relevance to assess how the study could contribute to program theory development. The type of data extracted is listed in Table 2. The relevancy was coded using four levels: 1 = very relevant, use of case management functions to connect patient or family to health, social and community services and supports; 2 =  very relevant to identifying critical community resources; 3 = very relevant, identifies barriers and facilitators to case management, 4 = very relevant, to local context but content not as relevant). Data from the articles was sorted by relevancy, only articles rated 1, 3 or 4 for relevancy were included for further analysis.

**Table 2:** Type of Data Extracted

1) Aim or purpose of the article	
2) Type of article: systematic review, quantitative study, qualitative study, commentary, theoretical, grey literature, other	
3) Category of outcome: name of quantitative outcome or qualitative finding	
4) Specific details on results/findings	
5) Relevancy: 1 = very relevant to case management functions, 2 = very relevant to identifying critical community resources, 3 = very relevant to identifying barriers and facilitators to case management, 4 = very relevant to local context but content not as relevant, 5 = interesting but not directly relevant, 6 = not that relevant	
6) If-then statements were used to help extract data in the form of explanatory accounts. Configuring if-then statements at the time of data extraction provided a way for the team to initiate new hypotheses, and informed program theory refinement in later stages.	

#### Defining critical community resources

The term critical community resources had been used in our initial program theories, but it had been poorly defined. This term needed further clarification to help focus our review. This led to an exploration into what community resources should be classified as critical. The first step was to explore the literature. The literature coded ‘2’ for relevancy (i.e., very relevant-identifying critical community resources) was used to identify a sample of studies (*n* = 61). Fifteen of the studies provided views of providers, patients, and families on what community resources were critical, and were relevant to our review. Data extracted from the studies was synthesized to develop themes. The studies did not provide enough evidence to determine which community-based resources were critical; however, there was evidence to support the essential resource of having family/friend caregivers and the need to tailor resources to what patients and their families need. In addition, the synthesis provided a list of 18 possible resources patients and families may want to consider when planning for end of life.

To supplement the synthesis, family advisors were asked to share their perspectives on which of these community resources they thought were critical. This methodology aligned with evidence from the literature that resources needed to be tailored to the needs of patients and their families. Family advisors were asked to identify their top four priorities from the list of 18 resources, and to include a rationale for their choices. This information was reviewed and synthesized by the lead researchers to establish a list of five critical community-based resources: 1) having a healthcare professional or assistant trained in end-of-life care, 2) being able to access someone trained in end-of-life care when patients transition home after discharge, 3) help with coordinating the available services and supports, 4) having resources available to help caregivers cope with stress and care for the patient, and 5) providing extra physical and psychological support for patients living alone. Two community resources aligned best with care early in the trajectory toward end of life and PPC. They were having practitioners trained in end-of-life-care and helping patients with the coordination of services and supports.

### Step 4: extracting and organizing data

Data extraction from the included articles was done using Excel by two reviewers, one of which was a research team lead (GW, LGB) and the second a research team member. Team discussions resolved any conflicts. A core team of three researchers went through the extracted data to help categorize and sort which articles contributed to elements of potential program theories: M (Resources), Contexts, M (Reasoning), Outcomes. From this process, specific evidence-based program theories were created and gaps in the literature were identified, this is what stimulated the second librarian assisted search and the researcher-initiated purposive searches described in Step 2. Six program theories evolved from the extracted data (see Additional File 3). The program theories reflected case management functions that needed to be implemented across a spectrum of care, these included having end-of-life conversations, planning for end-of-life, communicating plans, identifying community resources, and helping patients.

To ensure that data extraction was comprehensive and aligned with proposed program theories the most relevant articles were downloaded into NVivo 12 for final coding. Articles were coded both deductively and inductively. The deductive codes looked for program theory elements M (Resources), multi-level contexts (C), M (Reasoning), and outcomes (O), and inductive codes explored concepts that could inform our theory development. Examples of inductive codes that evolved were care coordination, collaboration, communication, tools to assess patient and family needs at end-of-life, and patient centred care.

### Step 5: Synthesising the evidence and drawing conclusions

The NVivo codes and associated text from the articles were critically reviewed. This review consisted of moving iteratively between the extracted text and potential program theories to understand the place of, and relationship between, each M (Resources), Contexts, M (Reasoning), and Outcomes within the program theory. This analytical process was informed by feedback from our non-academic partners to develop and refine potential program theories that could be supported by the evidence and were plausible. Through this process the research team established six criteria the refined program theories needed to meet. The refined program theories needed to: 1) address the goal of our review, 2) focus on patients and their families at the beginning of the palliative care trajectory when services are provided in primary care, 3) identify M (Resources) that facilitated case management functions, 4) describe M (Reasoning) that were Primary Care Practitioners’ (PCPs) internal reactions, 5) align with identified critical community resources relevant to primary care, and 6) reflect existing or potential (possible, projected) contexts relevant to health system partners.

The majority of extracted evidence supported the first two of our initial six program theories: having end-of-life conversations and planning for end-of-life. These theories reflected case management functions associated with patient/family identification, assessment, and planning within primary care practices. It was not feasible for the current review to explore all six theories in sufficient depth. As a result, the first two theories became the focus of this review. The rationale for choosing these theories was that there was a perceived readiness within the health system to support initiatives focused on helping primary care implement a palliative approach to care. Additionally, in order for primary care to initiate actions that would connect patients and their families to critical community resources, practitioners first needed to have a conversation, assess needs related to end-of-life, and create plans based on patients’ and family caregivers’ identified needs.

## Results

**Fig. 1:**
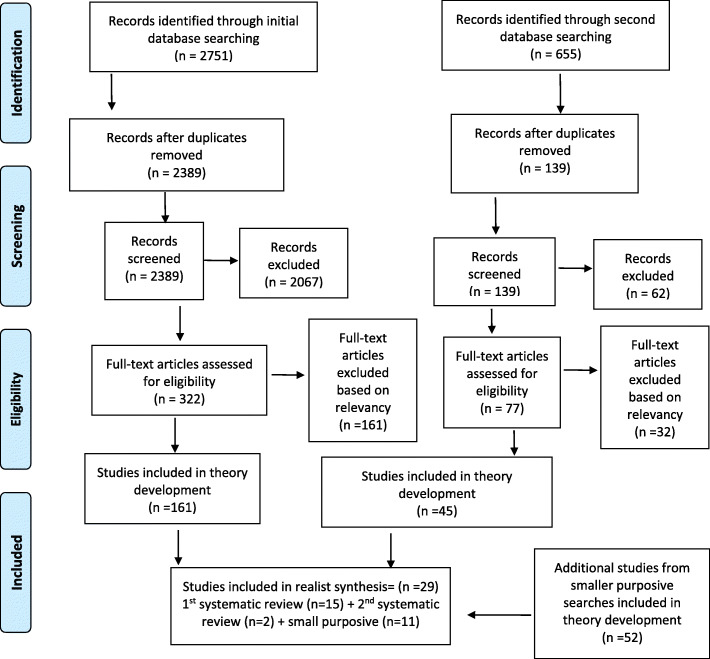
PRISMA diagram

The Prisma diagram (Fig. 1) summarizes results from the systematic and purposive searches. Program theory development was based on 258 included studies. This number was reduced to 77 studies when data was sorted for relevancy and only articles rated 1, 3 or 4 for relevancy were included for further analysis. This number was further reduced to 69 studies when the data extraction was reviewed to determine how each article contributed to our six initial program theories. These 69 studies were downloaded into NVivo 12 for final coding. The final coding and reducing the number of theories from six to two left 29 included studies as being relevant to the review.

There were six mixed methods studies, fifteen qualitative studies, one review of systematic reviews, four systematic reviews, and one theoretical case study. These studies came primarily from the initial systematic review (*n* = 15) and the purposive/citation searches (*n* = 11), with only a few coming from the second systematic search (*n* = 2). The second systematic review captured many of the studies in the initial systematic search and were deleted as they were duplicates. Studies were categorized in the levels of evidence described in the research pyramid [37], this information along with further details on the methods, aims, and outcomes assessed are in Table 3. The evidence from these articles was consolidated into two final program theories: Theory 1: Making end-of-life discussions comfortable; and Theory 2: Creating plans that reflect needs & values. For our two program theories, M (Resources) reflected resources that facilitated patient/family identification, assessment, and planning in primary care, and M (Reasoning) were reactions of PCPs to using these resources in different contexts. Several articles contributed to both theories, such as those describing contexts where PCPs did not feel they had time to initiate end-of-life conversations or to conduct advance care planning. Also, the two theories sometimes reflected a sequential causal chain, where the outcome of theory 1 affected the context for theory 2. For example, if end-of-life conversations had not been initiated, then advance care planning was unlikely to happen; or if end-of-life conversations were initiated by assessing the needs of patients and their families, this may present an opportunity to do advance care planning. Table 4 indicates how much each included article contributed to the program theory components by cross-referencing the article with program elements. Additional File 4 presents data extracted from each article to support or refute each program theory.

**Table 3:** Included Studies

Study ID; theory contribution; search	Aim/purpose of the article	Type of article & methodology	Outcomes assessed or qualitative study information	Results/findings from study	Level of Evidence
Aoun, 2015 [34]; Theories 1&2, initial systematic search	To investigate the impact of using a carer support needs assessment tool to identify and address support needs in end of life home care, on family caregiver outcomes	Quantitative Study. Stepped-wedge cluster non-randomised trial	Caregiver strain and distress as measured by subscales of the Family Appraisal of Caregiving Questionnaire (FACQ-PC). Secondary outcomes were caregiver mental and physical wellbeing as measured by SF-12 and caregiver workload as measured by caregiver assistance with Activities of Daily Living	Using the carer support needs assessment tool was associated with a significant reduction in Caregiver Strain, an improvement in the SF12 Mental Component Score, (it was not statistically significant after adjusting for covariates, *p* = 0.67). Both groups showed a worsening on the SF12 Physical Component Score (PCS) over the two time points, but these differences were not significant	Experimental research, Level 3 controlled trial
Bainbridge, 2016 [35]; Theories 1&2; initial systematic search	To determine which components of in-home end-of-life care programs are most commonly associated with better quality, effectiveness, or cost outcomes than usual care	Review of systematic reviews. Review methodology	Components of in-home end-of-life care programs are most commonly associated with better quality, effectiveness, or cost outcomes than usual care	On average, each program contained 11 components; the six most common were linkage with acute care, multidisciplinary nature, end-of-life expertise and training, holistic care, pain and symptom management, and professional psychosocial support. Linkage, to around-the-clock availability, and customized care planning were most common to the nine interventions for which a significant cost reduction was reported. Programs that included linkage with other community providers or primary care tended to have positive outcomes in a high proportion of studies	Descriptive research, Level 1 systematic review
Bischoff, 2013 [36]; Theories 1&2; initial systematic search	To examine the relationship between advance care planning and the quality of end-of-life care, as measured by the rates of care consistent with pre-existing quality metrics	Quantitative Study. Combined administrative cost data with large cohort data-Medicare data with the Health and Retirement Study cohort	Quality of end-of-life care	Advance care planning is associated with decreased in-hospital death and increased hospice use. All components of advance care planning are associated with the end-of-life care received	Outcome research, Level 2 pre-existing group comparison
Blackford, 2012 [37]; Theory 2; second systematic search	To develop a service evaluation tool for an advance care planning model implemented in community palliative care	Mixed methods Study. Multisite action research approach with three community palliative care services located in Victoria, Australia	﻿Qualitative and quantitative data collection used to develop the Advance Care Planning-Service Evaluation Tool (ACP-SET). This tool was designed to assist community-based palliative care services in Australia to establish a sustainable system-wide ACP Model relevant to their local context	The ACP-SET Advance Care identified advance care planning progress over time across three stages of Establishment, Consolidation and Sustainability within previously established Model domains of governance, documentation, practice, education, quality improvement and community engagement. The tool was used either as a peer-assessment or self-assessment tool to assist services to track their implementation progress as well as plan further change strategies. The ACP-SET was useful to managers in community palliative care. It provided a clear outline of service progress, level of achievement and provided clear direction for planning future changes. The ACP-SET enables practitioners in community palliative care to monitor, evaluate and plan quality improvement of their advance care planning model to improve end-of-life care	Descriptive research, Level 2–3 multiple case study; Qualitative research, Level 2 group qualitative research with more rigor
Blackford, 2013 [38]; Theory 1; second systematic search	The paper is associated with Blackford 2012. It presents findings of a study that aimed to address the question ‘How can palliative care nurses initiate and facilitate an ACP conversation in community palliative care practice?’	Mixed methods Study. Multisite action research approach with three community palliative care services located in Victoria, Australia	Baseline audit of staff and clients; analysed relevant documents (client records, policies, procedures and quality improvement strategies) pre-implementation and post-implementation and conducted nine key informant interviews	The audit identified that none of the nurses regularly spoke to clients about ACP. The most common reasons nurses articulated for the absence of ACP were a conviction that these discussions were the work of the social worker or counselor and the feeling that either ‘the training that they had wasn’t enough, or they just didn’t feel confident enough to bring up some of those subjects	Descriptive research, Level 2–3 multiple case study; Qualitative research, Level 2 group qualitative research with more rigor
Coventry, 2005 [39]; Theory 1; purposive search	To identify and evaluate potential decision-making tools and predictor variables that might aid clinicians to determine ≤6 months survival in older, non-cancer patients	Systemic review. Review methodology	Identification of potential decision-making tools and predictor variables to determine ≤6 months survival in older, non-cancer patients	Given the unpredictability of the nature of progressive life-threatening non- cancerous illnesses it is difficult to determine a specific cut-off point to refer patients to palliative care. Starting palliative care as early as possible, at any stage of the illness, along with curative measures would be beneficial for patients. There is a need to be aware of palliative care and commutate this with patients and their families	Descriptive research, Level 1 systematic review
De Vleminck, 2013 [40]; Theories 1&2; purposive search	To identify the perceived factors hindering or facilitating General Practitioners (GP) in engaging in advance care planning with their patients about care at the end of life	Systematic review. Review methodology	Perceived factors hindering or facilitating GPs in engaging in advance care planning	Initiation of advance care planning in general practice may be improved by targeting the practitioner’s skills, attitudes, and beliefs, but changes in health care organization and financing could also contribute. Understanding the barriers and facilitators is important to develop strategies promoting end-of-life conversations in Primary Care. For example, educational programs aiming to change attitudes or improve healthcare providers skills, and compensation policies	Descriptive research, Level 1 systematic review
Dingley, 2016 [41]; Theory 2; initial systematic search	To examine caregiver and nurse communication behaviors associated with caregiver activation during home hospice visits of patients	Qualitative Study Prospective observational design	Thematic analysis to develop codes for nurse communications	Caregiver communication that reflected activation included demonstrating knowledge regarding the patient and carer, describing care strategies, expressing opinions regarding care, requesting explanations of care, expressing concern about the patient, and redirecting the conversation toward the patient. Nurses responded to carers by providing education, reassessing the patient and care environment, validating communications, clarifying care issues, updating/revising care, and making recommendations for future care. Nurses prompted carer activation through focused care-specific questions, open-ended questions/statements, and personal questions	Qualitative research, Level 2 group qualitative studies with more rigor
Ewing 2016 [42]; Theories 1&2; initial systematic search	To examine practitioner perspectives on the impact, and mechanisms of action of the Carer Support Needs Assessment Tool in palliative home care	Qualitative Study. Practitioner accounts of implementation (interviews, focus groups, reflective audio diaries) plus researcher field notes	Patients’ experiences of being in receipt of the evolving and complex process of advanced care coordination within and between primary and secondary care settings in order to inform current debates on care coordination of patients toward	Existing approaches to identification of carer needs were informal and unstructured. Practitioners expressed some concerns, pre-implementation, about negative impacts of the Carer Support Needs Assessment Tool on carers and expectations raised about support available. In contrast, post-implementation, the Carer Support Needs Assessment Tool provided positive impacts when used as part of a career-led assessment and support process: it made support needs visible, legitimised support for carers and opened up different conversations with carers. The mechanisms of action that enabled the Carer Support Needs Assessment Tool to make a difference were creating space for the separate needs of carer, providing an opportunity for carer to express support needs and responding to carer self-defined priorities	Qualitative research, Level 2 group qualitative research with more rigor
Gallagher 2012 [43]; Theory 1; initial systematic search	To develop and evaluate a form with a brief overview of each patient’s plan of care for use in multidisciplinary team meetings and everyday clinical practice in the community	Qualitative research report on how a care team used a form to communicate. Discusses the development and evaluation of a form with a brief overview of each patient’s plan of care for use in multidisciplinary team meetings and everyday clinical practice in the community	Costs (of development) and evaluation feedback were used to improve the service and form	Costs in team hours are outlined; feedback to improve form; data collected on care outcomes: information about individual and team workloads on a month-by-month and annual basis. Data on referral patterns, number and place of deaths, and the number of end-of-life tools being used	Qualitative research, Level 4 qualitative study with a single case
Gardiner 2015 [44]; Theories 1&2; purposive search	To explore the evidence relating to transitions from curative care to palliative care	Systematic review. Review methodology	Review of the evidence relating to transitions to palliative care within a UK context	The reviewed studies examined patients and providers experiences with regards to transition to palliative care. Findings show poor communication, mixed messages, unrealistic information resulted in patients’ unrealistic expectations (mentioned by healthcare providers) and feeling of fear and uncertainty about the palliative care. Another interesting finding is the concern about the continuity of care. All these imply the necessity of having clear communication with patients/ families, as well as interprofessional and intra-professional ones	Descriptive research, Level 1 systematic review
Holdsworth, 2011 [45]; Theories 1&2; initial systematic search	To identify issues around discussing and recording preferences on place of death from the perspective of hospice patients, carers, and hospice community nurse specialists	Qualitative Study. Focus group and interviews across three settings.	Thematic analysis cross referenced emerging themes between all groups	Important to build relationships with patients and carers, this makes it easier to talk about dying. The timing of discussions on dying was also thought to be important. The patient’s understanding of their prognosis was felt to be a precondition for the discussion by health professionals. Patients may not be aware of the practicalities around planning care, but having a discussion focused on service provision and availability may be a useful and acceptable step toward a discussion on their end-of-life wishes	Qualitative research, Level 2 group qualitative studies with more rigor
Howard, 2018 [46]; Theories 1&2; purposive search	To identify barriers to and enablers of advance care planning perceived by physicians and allied health professionals in primary care	Quantitative Study. Cross-sectional, self-administered survey in Ontario, Alberta, and British Columbia, Canada	The primary outcome was rating of the magnitude of each barrier by respondents using a 7-point scale from 0 to 6 (not at all, very little, a little, a moderate amount lot, a great deal, an extreme amount)	Physicians rated 4 barriers with a mean score of 3 (a moderate amount) or higher: insufficient time, inability to electronically transfer the patient’s advance care plan, decreased interaction with patients owing to transfer of care, patients’ difficulty understanding the limitations and complications of life- sustaining therapies. Allied Health Professional identified 12 barriers with a mean score of 3 or higher. Three were the same as for physicians: Inability to electronically transfer the patient’s advance care plan, decreased interaction with patients owing to transfer of care, patients’ difficulty understanding the limitations and complications of life-sustaining therapies. They also rated a lack of knowledge of ACP significantly higher compared with physicians (P < .001), and rated lack of time significantly lower (*P* < .001). In the qualitative comments five themes emerged to overcome barriers: public engagement, health care provider attitudes, creating capacity for primary care providers, integration of ACP into the workflow, and system and policy support	Descriptive research, Level 2 correlational study and Qualitative research, Level 2 group qualitative study with more rigor
Jacobsen, 2017 [47]; Theories 1&2; purposive search	To describe a dual framework that focuses on living well while acknowledging the possibility of dying to help outpatient clinicians working with seriously ill patients hold both possibilities	Theoretical article based dialectical behaviour therapy and acceptance/commitment therapy that includes individual case studies. This article describes how trained clinicians can use a dual framework to help patients navigate a developmental process in which patients maintain a focus on living well while simultaneously learning to consider and talk more explicitly about the possibility of dying	They discuss a dual framework and offer a definition, then they describe (1) how to start using the dual framework with patients, (2) how to use the framework to help patients define for themselves what it means to live well, and (3) how to use the framework to gently encourage patients to acknowledge and tolerate discussions about the possibility of dying	The article describes the importance of building rapport, communication and providing a supportive environment for seriously ill patients to acknowledge their conditions, help them have a personalized definition of living well, acknowledge of the possibility of dying and being prepared for the future that developing an ACP could be one of those preparations. This stepwise framework helps clinicians start off and maintain difficult conversations with patients. Starting to Use the Dual Framework involves two steps: First, clinicians encourage a patient swinging between optimistic hopes and realism to consider both possibilities nearly simultaneously. In the second step, once the clinician has linked optimistic hopes with the illness, the clinician encourages the patient to expand his or her hopes to include living well. The subtle shift from pure optimism to living well allows the patient to be future oriented and hopeful for a possibly achievable outcome. Over time, the clinician and patient work together to define better how to live well	Descriptive research, Level 4 individual case studies
Johnston, 2009 [16]; Theory 1; purposive search	To find out what is known about how people experiencing end of life care manage their illness themselves, in the advanced stages of their disease	Systematic Review. Review methodology	Synthesized evidence o what is known about how people experiencing end of life care manage their illness themselves, in the advanced stages of their disease	Three main themes were identified from the literature that formed the outline of the literature review; interventions for end of life care; self-care behaviours used by patients; factors that prevent patients to self-care	Descriptive research, Level 1 systematic review
Jones 2014 [48]; Theories 1&2; purposive search	To explore providers’ opinions about adoption, implementation and maintenance of a patient decision aids (PtDA) designed for patients and families facing serious illness	Qualitative Study. Focus group data using an inductive interpretive approach to explore patterns and preliminary themes in the data	They analyzed provider and patient focus group data using an inductive, team-based, interpretive approach to explore patterns and preliminary themes in the data.	The study examined the perceptions of physicians and patients about PtDA as a tool to improve shared decision making. The findings showed a gap between patients and non-palliative care clinicians: “Clinicians were afraid the tool could be seen as a ‘death message’, abandonment and/or ‘giving up’ on the part of the provider”, but patients found it a useful tool that should be introduced to them earlier rather than later, “never too early”. Their findings indicate patients expect their provider needs to be better informed and make decisions when they are still well. Patients indicated that there needs to be an effective communication strategy about end-of-life throughout the community. The article’s findings sugget promoting interpersonal communication skills, community awareness, and knowledge and use of community resources	Qualitative research, Level 2 group qualitative research with more rigor
Kelley 2013 [49]; Theory 1; initial systematic search	To describe and organize caregiver pain management challenges faced by home hospice caregivers of cancer patients	Qualitative Study. Content analysis of secondary data	Recordings of caregiver interviews, to describe pain management issues	The six major themes identified in the analysis included Caregiver-Centric Issues, Caregiver Medication Skills and Knowledge Issues, End-of-Life Symptom Knowledge Issues, Caregiver Medication Skills and Knowledge Issues, End-of-Life Symptom Knowledge Issues, Communication and Teamwork Issues, Organizational Skill Issues, and Patient-Centric Issues	Qualitative research, Level 3 group qualitative with less rigor
Kramer, 2013 [50]; Theory 1; initial systematic search	To understand what social-workers do and their roles in providing end-of-life care to low-income older adults with multiple comorbid chronic conditions in a community- based managed care program, from multiple stakeholder perspectives (i.e., older adults, family caregivers, team members, and social workers themselves)	Mixed Methods Study. Multimethod longitudinal case study	Survey reports of needs addressed by social workers for deceased older adults, focus groups with interdisciplinary team members, and in-depth interviews with older adults and their family caregivers. Thematic conceptual matrix was developed to detail distinctive social work roles that address divergent needs of older adults, family, and team members	Distinctive perceptions of social workers’ roles were identified for the different stakeholder groups (i.e., older adults, family caregivers, team members, and social workers). Older adults identified seven primary roles that social workers have in helping them: ensure that basic needs are met; provide meaningful caring relationship; complete organization tasks; help make informed decisions; prepare for future and for death; tackle problems; and watch over older adult. Three primary roles that social workers were perceived to have in helping the family were: provide information, provide emotional support, and take burden off. Family members acknowledged all of the roles reported by the older adult, but they identified six additional roles in helping the older adult (i.e., provide intellectual and social stimulation, address grief and bereavement, provide emotional support, facilitate transitions, facilitate independence, and serve as central care mangers), and three additional roles that social workers play in helping the family (i.e., facilitate transitions, facilitate family communication, and prepare family for future and for death). Family members reported tremendous appreciation and trust they felt in reliance on social workers who had cultivated meaningful, long-term caring relation- ships with the elder	Qualitative research, Level 2 group qualitative research with more rigor
Le, 2017 [51]; Theory 1; initial systematic search	To determine General Practitioner (GP) needs when providing home-based palliative care in collaboration with existing palliative care services	Quantitative Study. Online survey	Outcomes were to determine knowledge, skills and confidence of GPs in providing community-based palliative care	Of the 56 respondents, 82% reported that they were involved in palliative management of at least one cancer patient in the previous year. A significant number of GPs (31%) lacked confidence in providing this care because of patient complexity, inadequate training and insufficient resources. Other barriers included poor communication from specialists and treating teams. Factors facilitating provision of home-based palliative care were community palliative care services and links to hospital-based palliative care teams	Descriptive research, Level 2 correlational study
Linderholm, 2010 [52]; Theory 1; initial systematic search	To explore how the family/friend caregiver of a dying relative in palliative home care experienced their caring role and support during the patient’s final illness and after death	Qualitative Study. A hermeneutic approach was used to analyze the data	Fourteen family members were selected in 4 primary health care areas in Sweden. Data were collected using open, tape-recorded interviews. A hermeneutic approach was used to analyze the data	The findings revealed that being a family/friend caregiver was natural when a relative became seriously ill. More or less voluntarily, the family member took on a caring role of control and responsibility. The family/friend caregiver felt left out and had feelings of powerlessness when they did not manage to establish a relationship with the healthcare professionals	Qualitative research, Level 2 group qualitative research with more rigor
Lum, 2017 [53]; Theories 1&2; purposive search	To explore whether participation in an Advance Care Planning- Group Visit (ACP-GV) intervention for older adults increased documentation filed within the health care system of either surrogate decision maker(s) or goals for medical care in an ACP document compared with before the intervention	Mixed Methods Study. The ACP-GV intervention was integrated into existing workflows of primary care clinics. Older adults (> 65 years) in primary care participated in a 2-session ACP-GV intervention that promoted group dynamics, peer-based learning, and goal setting. … facilitated by a physician and social worker pair who used a facilitators’ guide to conduct a semi-structured group interaction	Charts were reviewed at baseline, 3 months, and 12 months for documentation of decision makers and ACP forms. Patients’ reasons for participating was described through analysis of transcripts	The ACP-GV intervention significantly increased ACP documentation of surrogate decision makers and goals for future medical care among older adults in primary care clinics	Descriptive research, Level 2 correlational study and Qualitative research, Level 3 group qualitative study with less rigor
OudeEngberink, 2017 [54]; Theory 1; initial systematic search	To determine how French General Practitioners (GP) provide palliative care in at-home settings, what their needs may be, and what skills and resources they mobilize for these interventions	Qualitative Study. Phenomenological questioning	An interview guide including phenomenological questioning focused on the GPs lived experience in providing palliative care	Offering palliative care was perceived by GPs as a moral obligation. They felt vindicated in a process rooted in the paradigm values of their profession. This study results in two key findings: firstly, their patient-centred approach facilitated the anticipatory discussions of any potential event or intervention, which the GPs openly discussed with patients and their relatives; secondly, this approach contributed to build an “end-of-life project” meeting patients’ wishes and needs. The GPs all shared the idea that the end-of-life process required human presence and recommended that at-home care be coordinated and shared by multi-professional referring teams. Theme 1: Palliative care represents another dimension of care: the transition from a disease-centred curative paradigm to a patient-centred multi-dimensional support and end-of-life quality paradigm. Theme 2: GPs’ patient-centered approach combines duty as a human being with professional and personal values. Theme 3: Discussing and anticipating potential events allows GPs to collaboratively devise “end-of-life projects” with their patients. Theme 4: Organizing human presence around the patient by sharing the caregiving amongst a multi-disciplinary team	Qualitative research, Level 2 group qualitative research with more rigor
Sanders, 2008 [55]; Theories 1&2; purposive search	To examine the impact of incorporating ACP within a self-management intervention	Qualitative Study. Qualitative interviews with participants enrolled in an RCT evaluation study of the Expert Patients Program versus an Expert Patients Program with for those HIV positive	The principal aim of the qualitative component of the evaluation was to provide a deeper understanding of the lives of people living with a chronic condition and the complexities and implications of planning for end of life care	The study indicates that people are different in their experiences with their health issues, as such in introducing new concepts such ACP one size doesn’t fit all. It is important to be aware of the context for talking about planning for death, building relationships, and providing adequate and correct information/expectations	Qualitative research, Level 2 group qualitative research with more rigor
Seymour, 2010 [56]; Theories 1&2; initial systematic search	To examine how community nurses working in palliative care understand ACP and their roles within ACP and to identify factors surrounding community nurses’ implementation of ACP and nurses’ educational needs	Qualitative Study. Community nurses took part in 6 focus group discussions about experiences of providing end-of-life care and views about ACP. Data analysed using a constant comparison approach	Experiences and views and collaborative interpretation of the focus group data and identification of key themes and developing ideas about educational resources for ACP	Nurses understood ACP to be an important part of practice and to have the potential to be a celebration of good nursing care. Nurses saw their roles in ACP as engaging with patients to elicit care preferences, facilitate family communication and enable a shift of care focus towards palliative care. They perceived challenges to ACP, including: timing, how to effect team working in ACP, the policy focus on instructional directives which related poorly to patients’ concerns; managing differences in patients’ and families’ views. Perceived barriers included: lack of resources; lack of public awareness about ACP; difficulties in talking about death. Nurses recommended the following to be included in education programmes: design of realistic scenarios; design of a flow chart; practical advice about communication and documentation; insights into the need for clinical supervision for ACP practice. Potential for community nurse (or equivalent) to have a key role in facilitating a process of ACP which has the potential to improve the quality of end-of-life care that patients receive. Identified challenges and barriers that must be addressed for this function to work	Qualitative research, Level 2 group qualitative research with more rigor
Sudore, 2017 [30]; Theory 1; purposive search	To develop a consensus definition of ACP for adults	Qualitative Study. Modified Delphi. Convened Delphi panel of multidisciplinary, international ACP experts consisting of 52 clinicians, researchers, and policy leaders from four countries and a patient/surrogate advisory committee	Conducted 10 rounds using a modified Delphi method and qualitatively analyzed panelists’ input. Panelists identified several themes lacking consensus and iteratively discussed and developed a final consensus definition of ACP	Definition of ACP: “Advance care planning is a process that supports adults at any age or stage of health in understanding and sharing their personal values, life goals, and preferences regarding future medical care. The goal of advance care planning is to help ensure that people receive medical care that is consistent with their values, goals and preferences during serious and chronic illness.”	Qualitative research, Level 2 group qualitative research with more rigor
Thomas, 2010 [57]; Theory 2; initial systematic search	To determine how family/friend caregiver needs are currently assessed and what level of support is available to family/friend caregivers in three home-based palliative care services within Australia, identify areas for improvement of support to family/friend caregivers, and explore the barriers to offering carer support	Mixed methods Study. A case study using focus groups and file audit patients who had been discharged within the past 6 months were conducted at two metropolitan and one regional home-based palliative care service in Australia	This is a multiple case study that looked at several sites, both metropolitan (M) and regional (R) Each regional centre had a distinct model of care. One used a case management model of care whereas a second provided consultation only and relied on the General Practitioner (GP) and the local district nursing service to provide the majority of ongoing care. Staff from all disciplines were invited to participate in order to ensure that a comprehensive range of perspectives were explored. Files were audited to see how often and in what manner carers’ care planning strategies were mentioned in the patients’ care plans and progress notes by a criteria-based system	In general, the findings were the following. These palliative care sites reported substantially different levels of services provided to family/friend caregivers and also reported multiple barriers to providing services for family/friend caregivers. Only one site had a formal structured procedure to assess family/friend caregivers needs and none of the sites used a separate family/friend caregivers care plan or offered a structured intervention to assist family/friend caregivers with their role. Family meetings were offered infrequently by most sites. A number of barriers to supporting family/friend caregivers were highlighted including lack of resources, and areas for improvement were also suggested by health professionals from the sites	Descriptive research, Level 2–3 multiple case study; Qualitative research, Level 2 group qualitative research with more rigor
Ventura, 2014 [58]; Theory 1; initial systematic search	To describe, evaluate and summarise the literature on the unmet needs of palliative home care patients and carers	Systematic review. Review methodology- using qualitative and quantitative studies	Seven databases were searched to find empirical studies on the self-reported unmet needs of palliative home care patients and carers. Nine qualitative studies, three quantitative studies and three mixed-design studies were identified	The most frequently reported unmet need was effective communication with health-care professionals, the lack of which negatively impacted on the care received by patients and carers. Physical care needs were met, which indicates that the examined palliative home care services were delivering satisfactory care in this domain but lacking in other areas. Some of the needs were respite care, information and advice, financial assistance, assistance with household tasks, emotional support, help with personal care and technical tasks	Descriptive research, Level 1 systematic review
Wharton, 2015 [59]; Theory 1; initial systematic search	To describe a pilot home-based primary care program (HBPC), which is a longitudinal home care in which primary care is delivered in the home aimed at maintaining independence and function and preventing read- mission of patients to the acute care setting. The goal was to increase palliative care knowledge and collaboration among providers and to systematically identify chronic multimorbid home care patients who would benefit from focused discussion of potential palliative care needs	Mixed Methods Study. Retrospective pre-post format. Solicited feedback on training from nurses. The program aimed to manage complex, chronically ill patients and improve the long- term health outcomes of participants with complex comorbid conditions while helping to contain health care costs	The model included—education, relationship-building between teams, and implementation of a validated screening tool to identify patients for targeted discussion. HBPC teams were made up of a physician, several nurses, a clinical psychologist, a physical therapist, a dietician, a social worker, and administrative support staff. Services could include blood draws, regular health checks and nursing care, functional mobility, physical therapy assessments and interventions, dietary consultation and education, psychological services for both client and family caregivers, and resource assistance, among other things. The pilot used the End of Life Nursing Education Curriculum (ELNEC) national PC curriculum. To address patient screening goals the PPS1 assessment tool was used as a template in the electronic medical record. The cut-off score of 40% or less, indicating a low-functional status, nutritional compromise, and possible altered cognition	ELNEC training, including both HBPC team members and members of other primary care teams in the hospital system was uniformly well received by the participants, with active learner participation and interaction prompted by relevant case discussions embedded within the ELNEC curriculum. Final course evaluations detected a substantial improvement between the learners’ self-rated knowledge of end-of-life care prior to the course. Findings indicated participants had increased confidence in their ability to use palliative knowledge in the course of their jobs. Nursing staff reported that they did not feel that the PPS was burdensome, although several were opposed to any change in their workload at all. The PPS might not be the best choice of instrument for a chronic, multi- morbid care population. There was some concern by team nurses that the PPS tool was not sensitive enough to conditions such as severe cognitive impairment, traumatic brain injury, or other chronic illnesses or disabilities, including accounting for an amputee status. Although minimal, the implementation of an additional screening tool did add to nursing time and effort. It was a challenge to ensure that the scores were entered in a consistent place in the medical record to enable easy access for review	Descriptive research, Level 2 correlational study and Qualitative research, Level 2 group qualitative study with more rigor
Wittenberg-Lyles,2011 [60]; Theory 1; purposive search	To determine which communication characteristics influenced early palliative care and how these shaped the patient’s and family’s healthcare experience and highlight issues with communication characteristics to inform the instrumental role of nurses	Qualitative study. A grounded theory approach	The researcher constantly compared field notes to the interview transcripts. Second, staff and interviewee feedback were collected, and themes were discussed and adjusted according to the feedback. Data was categorized by themes	Five communication characteristics were identified in the observations and interviews: (a) curative-only approach and the diagnosis, (b) the structure and communication of medical care, (c) productive experiences (open awareness), (d) embraced opportunities to plan for end of life, and (e) community. Building rapport, and having communication with patients about their conditions, and what may come up prepare patients for the future. These characteristics along with interprofessional communication, provided “a community of professionals” that helped patients move through the illness trajectory with comfort and trust. Findings indicate embedding palliative care into patients care as early as possible	Qualitative research, Level 2 group qualitative research with more rigor

**Table 4:** **Contribution of each article to the program theories**

	Program resources			Policies or population needs			Practice settings		Practitioner capacities and reactions			Outcomes	
	Theory 1a: Using tools or frameworks	Theory 1b: Learning to improve end-of life communications	Theory 2: Creating plans that reflect needs & values	Lack of resources	Patient population perceptions	Communication of plans	Lack of time or changing practice routines	End of Life Care culture	Creation of space trusting relationships	Discomfort with End of Life	Prior training, education, or disciplinary backgrounds	Better provider/caregiver communication	Improved care of patient and caregivers
Percent contribution to program theory	25%	46%	32%	25%	32%	4%	25%	14%	54%	21%	39%	36%	46%
Study ID													
Aoun, 2015	1						1				1	1	
Bainbridge, 2016			1	1		1							1
Bischoff, 2013			1		1							1	1
Blackford, 2012			1	1	1								
Blackford, 2013		1									1		
Coventry, 2005	1									1	1	1	
DeVleminck, 2013			1	1	1		1		1	1			
Dingley, 2016		1							1			1	1
Ewing, 2016	1								1			1	1
Gallagher, 2012	1												
Gardiner, 2015		1			1								1
Holdsworth, 2011		1			1				1		1	1	1
Howard, 2018			1	1	1		1				1		
Jacobsen, 2017	1								1			1	1
Johnston, 2009		1			1				1				1
Jones, 2014	1			1	1		1			1		1	
Kelley, 2013		1								1			1
Kramer, 2013		1									1		1
Le, 2017		1					1				1		
Linderholm, 2010		1						1	1				
Lum, 2017			1		1			1	1				1
OudeEngberink, 2017		1							1	1	1		
Sanders, 2008			1	1					1		1		
Seymour, 2010			1	1				1	1	1	1	1	1
Sudore, 2017			1						1				
Thomas, 2010		1							1				
Ventura, 2014		1					1						
Wharton, 2015	1						1	1	1				
Wittenberg-Lyles, 2011		1							1			1	1

### Theory 1: making end-of-life discussions comfortable

If PCPs use tools to assess needs of patients and their families nearing end-of-life, it can provide practitioners with an opportunity to discuss and identify end-of-life issues without specifically discussing death. Also, if PCPs receive training on how to better communicate, it can increase their confidence for initiating end-of-life discussions. In positive contexts the use of tools and having training can improve the reactions of practitioners M (Reasoning) to engaging patients and families in end-of-life conversations. This can improve communications within primary care teams and with patients and their families to improve patient and their family to planning for end-of-life (O). For theory 1, two categories of M (Resources) were identified from the literature.

**Fig. 2:**
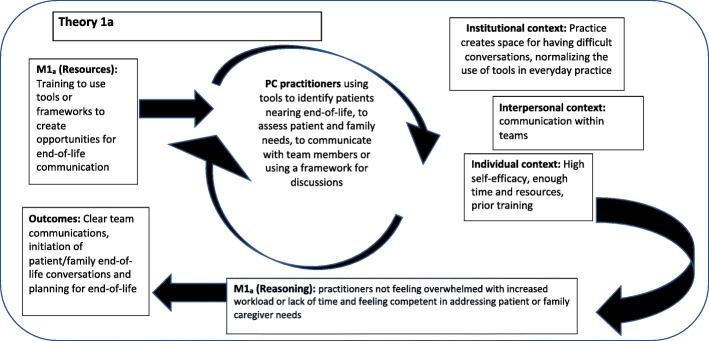
Theory 1a

**Fig. 3:**
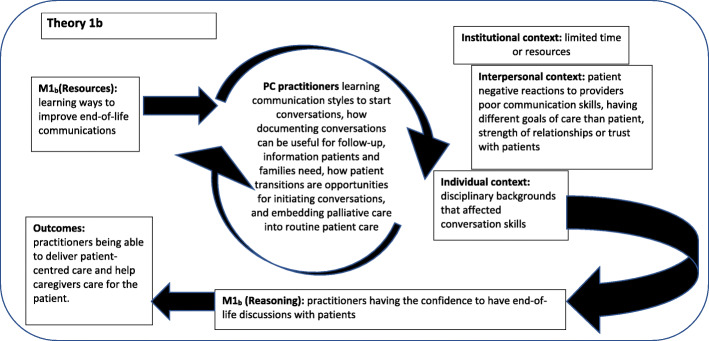
Theory 1b

a) Using Tools or Frameworks (Fig. 2 and Additional File 4). The first category of M (Resources) for theory 1 was using tools or frameworks. M1_a_ (Resources). The literature identified using tools or discussion frameworks as beneficial to: identifying patients nearing end-of-life [38], assessing patient and family needs [39–41], facilitating team communications [42], and structuring end-of-life discussions [43, 44]. Contexts (1_a_). The identified program resources interacted with various contexts such as: time or resources [39–41], practitioner self-efficacy [38], prior training [39, 45], creating spaces (i.e., safe spaces and opportune moments) for difficult conversations to take place [43, 45], and routine use of tools in everyday practice [41, 45]. The M1_a_ (Resources)-Context interaction affected M1_a_ (Reasoning) such as: practitioners feeling overwhelmed with increased workload and lack of time [39, 41], or alternatively feeling competent in addressing patient or family caregiver needs [40, 43, 45]. Positive reactions facilitated Outcomes (1_a_) such as: clear communications within teams [42], initiating conversations with patients and their families [38, 40], and improved patient and family abilities to plan for end-of-life [39–41, 43].

b) Learning to improve end-of life communications. (Fig. 3 and Additional File 4) The second category of mechanisms for theory 1 was improving communication skills to discuss end-of-life. M1_b_ (Resources). Resources that supported PCPs initiating and engaging in constructive end-of-life conversations were: learning communication strategies for initiating conversations [46–49], using documented conversations for future follow-up [50], understanding what information patients and families needed [16, 51, 52], identifying patient transitions as opportunities for initiating conversations [53, 54], and embedding palliative care into routine patient care [52]. Contexts (1_b_). The identified M1_b_ (Resources) interacted with contexts such as: patient negative reactions to providers’ with poor communication skills [16, 51, 55–57], limited time or resources [46, 50, 51, 53, 54, 58], strength of relationships or trust with patients [48, 50, 51, 57], providers having different goals of care than patients [16, 50], and disciplinary backgrounds that emphasized conversation skills [49, 52–54, 57]. The interactions between M (Resources) and contexts affected M1_b_ (Reasoning) such as PCPs having the confidence to have end-of-life discussions with patients [54]. This reaction ultimately affected Outcomes (1_b_) such as: practitioners supporting caregivers to care for the patient [46], and delivering patient-centred care [54]. Furthermore, these led to longer-term outcomes such as: better preparation of patients and families for end-of-life and bereavement [16, 48, 50, 52, 57], and fewer aggressive life-sustaining medical interventions near death [52].

### Theory 2: creating plans that reflect needs & values. (Fig. 4 and Additional File 4)

If PCPs use tools to assess needs, or have others assess needs, this provides an opportunity to create a plan for the future that reflects what patients and families value. Planning with patients and their families can improve their ability to anticipate end-of-life events and can lead to positive patient end-of-life and family bereavement outcomes. For theory 2, only one category of M (Resources) was identified: facilitators for conducting advance care planning.

**Fig. 4:**
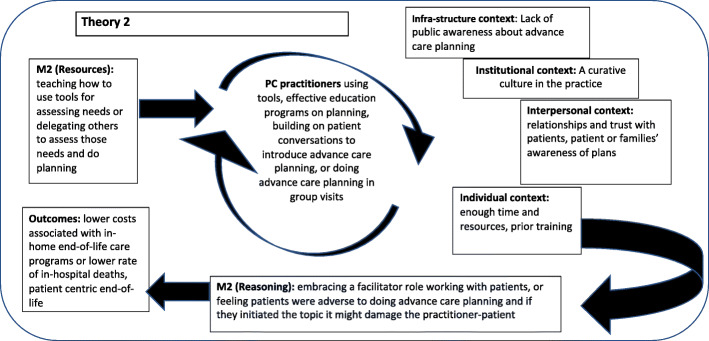
Theory 2

Facilitators for conducing advance care planning. M2 (Resources). Literature identified facilitators to conducting advance care planning such as: using tools [59], effective education programs for practitioners [60], building on patient conversations to introduce advance care planning [44], or doing advance care planning in group visits [61]. Contexts (2). These M2(Resources) interacted with contexts such as: limited time and resources [44, 60, 62], strength of relationships and trust with patients [60, 62], lack of public awareness about advance care planning [44], a curative culture in the primary care practice [44], and differing disciplinary backgrounds [44]. These contexts affected PCP readiness for advance care planning [44]. Interactions between the M (Resources) and contexts impacted M2 (Reasoning) of PCPs embracing a facilitator role where they worked along with patients [33, 61], or conversely practitioners feeling patients were adverse to doing advance care planning, and if they initiated the topic it might damage the practitioner-patient relationship [62]. These reactions ultimately affected whether the PCP engaged in advance care planning with patients and families; which could subsequently lead to long-term Outcomes [2] such as: reduction of costs associated with in-home end-of-life care programs and patient centric end-of-life care planning that included linking primary care with community resources [63]. In addition, advance care planning was found to be associated with a lower rate of in-hospital deaths [64].

Evidence from the articles demonstrated how implementing resources into supportive contexts could facilitate conversations that identify patients earlier in their trajectory toward end-of-life, creation of patient and family centric plans, and implementation of planned care.

## Discussion

The goal of the realist synthesis was to synthesize evidence on how multi-level contexts and mechanisms affect the implementation of the case management functions of patient identification, assessment, and planning; specifically as they relate to initiating end-of-life conversations, assessing patient and caregiver needs, and patient/family centred planning in primary care practices to improve outcomes. In addition, the synthesis explored how these functions aligned with critical community resources identified by patients and their families dealing with end-of-life.

As well as the involvement of health system partners, this review incorporated views of family advisors with end-of-life care experiences to provide a family-centric viewpoint. The engagement of family advisors played an essential role in focusing our review on what families valued, and the feedback from health system partners ensured the findings and recommendations were potentially feasible to implement within the health system.

Two program theories evolved over the course of the review: 1) making end-of-life discussions comfortable, and 2) creating plans that reflect needs and values. Some M (Resources) were relevant to both theories such as using tools or frameworks or learning how to take advantage of opportunities where patients or families are more open to engaging in end-of-life discussions. Using tools or discussion frameworks to assess patient and family needs was associated with not only facilitating end-of-life discussions but also advance care planning [39–41]. The key was to identify needs as a way of introducing necessary end-of-life topics [43, 44] and to help structure or conduct advance care planning [59, 61]. Also relevant to both theories was learning how to recognize and identify opportunities when patients may be more aware of the need to plan for their end-of-life care [44], such as in contexts when patients are experiencing transitions [45, 53, 54, 62]..

As patients’ end-of-life needs change and transitioning between health care settings becomes more frequent, patients and their family caregivers may become more aware of the need to plan for end-of-life care. This review revealed PCPs need to recognize and use these transitions as opportunities to introduce and discuss necessary end-of-life topics. This was highlighted in an editorial that listed opening questions a PCP might use when changes or transitions occur as a way of exploring end-of-life issues [65, 66]. The use of general questions may address PCPs’ negative reactions to using tools. Alternatively, the use of tools, discussion frameworks, or general questions could all create opportunities for PCPs discussing end-of-life issues [39]. These strategic approaches could ensure patients’ and family caregivers’ values and needs are identified and reflected in advance care plans as they transition between health care settings [67, 68].

Two contexts that negatively affected PCPs’ abilities to initiate end-of-life conversations and conduct advance care planning were not having enough time or resources (e.g., additional staff or services) [39–41, 44, 60, 62]. These challenges may be due to PCPs’ prioritization of time. Regardless these contexts limit opportunities for initiating and engaging in important, yet time consuming, end-of-life conversations with patients and their families [43, 45]. They may also impede practitioners’ abilities to develop trusting relationships with their patients [60, 68]. M (Resources) that might catalyze changes in these negative contexts include: developing practice routines that create supportive spaces for patient’s experiences to emerge [43], building an organizational commitment to end-of-life care and improving team communication skills [69], and embedding the use of tools that identify end-of-life issues into practice routines [41, 45]. In addition, there is evidence that advance care planning could be introduced and discussed in group sessions rather than in one-to-one conversations with practitioners [61].

Providing PCPs with training on how best to communicate with patients and their families about end-of-life [46–49] continues to be an unmet need in primary care settings [70–72]. Our review found that PCP attitudes towards advance care planning were influenced by previous experience, education and training in communicating with patients during end-of-life care [39]. These attitudes can facilitate or hinder PCPs initiating end-of-life conversations and planning with patients and their family/friend caregivers [10, 47]. The Gold Standards Framework, one of the most widely known frameworks for how to organize community-based palliative care, recommends that training be targeted towards, and carried out with PCPs who have an interest in end-of-life care [73]. Practitioner readiness for end-of-life training might increase in contexts where the practice setting serves a large geriatric patient population [74], or when practitioners have a disciplinary background that emphasizes strong communication skills, such as social workers, and nurses in specialty clinical areas such as oncology [52, 75–79]. In addition, the inclusion of palliative care content and clinical practice experiences in health care provider curricula has been shown to increase health care providers’ confidence and skills in initiating end-of-life discussions and advance care planning with patients and family caregivers [80].

If PCPs facilitate conversations and planning for end-of-life, then patient, family, and health system outcomes can be improved. Patients and families may be better prepared for end-of-life events and bereavement [48, 50, 52, 75, 81], and health systems are less likely to use aggressive life-sustaining medical interventions before a patient’s time of death [52, 82], have lower rates of in-hospital deaths [64], and decreased health care costs [63]. In addition to practitioner-patient conversations and planning, it is important that end-of-life plans are clearly communicated across health and social sectors involved in implementing the patients’ care plan [63, 70, 83]. Intersectoral communication was not explored in this review, but it is an important element that enables links to community resources and improved outcomes.

The literature demonstrated the benefits of families being involved in end-of-life planning. Better communication between PCPs and families helps increase family members’ confidence around caring for patients in their usual place of residence [84]. Despite this benefit, it is important to recognize that families may not be prepared for, or desire to, care for the end-of-life patient at home [85]. Some families may not be comfortable with the role of providing direct physical care or medication management and prefer to offer social and emotional care at end-of-life. To make end-of-life caregiving at home a reality, family members have to genuinely want to provide care, rather than being pressured into providing it; they also need to feel they can get support from the healthcare system when needed.

There are limitations to our review. It focused on case management functions of client identification, assessment, and planning as they were relevant to interventions initiated in primary care practices. However, case management includes other functions such as: implementation of planned services, evaluations that periodically assess patient and family needs, and transitioning the patient into other services [13]. Although these remaining functions were not emphasized in our review, they are still important and are often embedded in primary care quality improvement processes.

Although our review did not highlight the infrastructure context, policies are needed at multiple levels in order to create health and social structures that will normalize and facilitate an upstream approach for palliative care. For example, the public health approach to palliative care advocates for policy development and implementation in the practice, health system, and societal contexts [86]. Societal contexts that embrace the public health approach to palliative care and align with the Compassionate Communities model [35] can catalyze social changes in attitudes that reinforce health system policies. Furthermore, health system and practice-level policies could support the intentional implementation of tools and frameworks, as well as the inclusion of primary care team members with disciplinary backgrounds or training suited to conducting end-of-life conversations and planning. These changes could improve earlier identification of patients’ need for palliative care services, including those with end stage chronic illnesses [87].

In the review process, family advisors identified five priorities for community resources. Our review was unable to provide evidence for all five priorities, but they remain critical. The evidence synthesized to develop our two program theories addressed the priorities of improving health practitioner training on end-of-life care, and to a lesser extent, improving coordination of services/supports and identification of programs and resources to help families. Coordination of care can be linked to the quality of communication amongst PCPs, patients and families, and within health care teams themselves. A precursor to identifying needed community resources is the assessment of needs. Using tools to facilitate communication, assess patient/family needs, and developing a plan could improve both coordination and identification of community resources for patients and families at end-of-life.

## Conclusion

Recommendations from our review were shared with our health system partners to inform current and future initiatives in PPC. However, future research in the form of a realist evaluation of initiatives identified in our review need to be completed to further test our theories. Conducting a realist evaluation would refine our initial theories and contribute to developing a middle-range theory that would be generalizable to a variety of contexts [88]. Additional research is also needed to determine how best to connect patients and families with community resources that can augment end-of-life care delivered in primary care settings.

In conclusion, the realist review method enabled an evidence synthesis on how multi-level contexts and mechanisms affect initiating end-of-life conversations, assessing patient and family caregiver needs, and patient-family centric end-of-life planning in primary care settings. The realist approach involved health system partners and family advisors, helping ensure our review remained grounded in the local context and identified outcome priorities relevant to patients’ and family caregivers’ values and needs for end-of-life care. The further development and testing of our initial program theories can be used to provide direction and inform changes in primary care practices, including policies and research initiatives that support an upstream palliative care approach to care that can improve outcomes for patients and their family caregivers at end-of-life.

## Supplementary Information

**Additional file 1.** Model of Program Theory.

**Additional file 2.** Search Terms.

**Additional file 3.** Six initial theories.

**Additional file 4.** Tables providing evidence for Theories 1a, 1b, and 2.

## Data Availability

The preliminary data extraction tables are extensive and are available from the corresponding author upon reasonable request. The final extracted data has been summarized in the tables and additional documents.

## Abbreviations

1_a_: Theory 1a
1_b_: Theory 1b
2: Theory 2
C: Contexts
C-M-O: Context-Mechanism-Outcome
M: Mechanism
M (Reasoning): Mechanism (Reasoning)
M (Resources): Mechanism (Resources)
M1_a_ (Resources): Mechanism for Theory 1a (Resources)
M1_a_ (Reasoning): Mechanism for Theory 1a (Reasoning)
M1_b_ (Resources): Mechanism for Theory 1b (Resources)
M1_b_ (Reasoning): Mechanism for Theory 1b (Reasoning)
M2 (Resources): Mechanism for Theory 2 (Resources)
M2 (Reasoning): Mechanism for Theory 2 (Reasoning)
O: Outcome
PPC: Primary Palliative Care
PCP: Primary Care Practitioner
RAMSES: Realist and Meta-narrative Evidence Syntheses—Evolving Standards
