# Supplementary material for: Engaging family caregivers and health system partners in exploring how multi-level contexts in primary care practices affect case management functions and outcomes of patients and family caregivers at end of life: a realist synthesis
Source: BMC Palliat Care. 2021 Jul 16;20:114. doi: 10.1186/s12904-021-00781-8 (PMC8285870; doi:10.1186/s12904-021-00781-8)
Supplement: Supplementary file 1 — Additional file 1. Model of Program Theory. [file 12904_2021_781_MOESM1_ESM.docx]

**Context: Infrastructure**

**Health and Social Policies and Resources**

**Public Health Approach to Palliative Care**

**Case Management Competencies**

**Culture, Implementation Readiness, Structural characteristics, Communication**

**Context: Institutional**

**Implementation Evaluation**

**Transition**

**Identification Assessment Planning**

**Identification and assessment of patient and family needs**

**Context: Individual Capabilities**

**Context: Interpersonal**

Relationships

**Case Management Functions**

**Planning and help navigating**
