# Supplementary material for: Engaging family caregivers and health system partners in exploring how multi-level contexts in primary care practices affect case management functions and outcomes of patients and family caregivers at end of life: a realist synthesis
Source: BMC Palliat Care. 2021 Jul 16;20:114. doi: 10.1186/s12904-021-00781-8 (PMC8285870; doi:10.1186/s12904-021-00781-8)
Supplement: Supplementary file 2 — Additional file 2. Search Terms. [file 12904_2021_781_MOESM2_ESM.docx]

**Additional File 2: Search Terms**

1. **Search terms for Initial systematic search strategy for identification of studies run March 2017.**

**Databases searched/resources consulted:**

- Ovid MEDLINE(R) Epub Ahead of Print, In-Process & Other Non-Indexed Citations, Ovid MEDLINE(R) Daily and Ovid MEDLINE(R)
- CINAHL with Full Text

MEDLINE Search Strategy

*Ovid MEDLINE(R) Epub Ahead of Print, In-Process & Other Non-Indexed Citations, Ovid MEDLINE(R) Daily and Ovid MEDLINE(R), 1946-present*

1. ((home or home based) adj (palliative or end of life or terminal or hospice)).tw.

2. ((community or community based or community centered or community centred) adj (palliative or end of life or terminal or hospice)).tw.

3. ((home or at home or home based) adj (care or treat* or manage* or intervention*) adj3 (chronic or life limiting or disease or cancer or high symptom burden)).tw.

4. ((community or community based or community centered or community centred) adj (care or treat* or manage* or intervention*) adj3 (chronic or life limiting or disease or cancer or high symptom burden)).tw.

5. 1 or 2 or 3 or 4

6. framework*.tw.

7. model*.tw.

8. theor*.tw.

9. program*.tw.

10. structure*.tw.

11. support*.tw.

12. resource*.tw.

13. deliver*.tw.

14. review*.tw.

15. 6 or 7 or 8 or 9 or 10 or 11 or 12 or 13 or 14

16. 5 and 15

| ***Database*** | ***Interface*** | ***Dates*** | ***Results*** |
| --- | --- | --- | --- |
| MEDLINE | Ovid | 1946-present | 760 |
| CINAHL with Full Text | EBSCOhost | 1982-present | 1991 |
| **Total** | | | **2751** |
| **Duplicates removed** | | | **362** |
| **De-duplicated total** | | | **2389** |

1. **Second systematic search strategy for identification of studies** **on key topics** **run August 2018**

| 1 | (home adj (palliative or end of life or terminal or hospice)).ti,ab. |
| --- | --- |
| 2 | (home based adj (palliative or end of life or terminal or hospice)).ti,ab. |
| 3 | (community adj (palliative or end of life or terminal or hospice)).ti,ab. |
| 4 | (community based adj (palliative or end of life or terminal or hospice)).ti,ab. |
| 5 | (community centered adj (palliative or end of life or terminal or hospice)).ti,ab. |
| 6 | (community centred adj (palliative or end of life or terminal or hospice)).ti,ab. |
| 7 | (home adj (care or treat* or manage* or intervention*) adj3 (chronic or life limiting or disease or cancer or high symptom burden)).ti,ab. |
| 8 | (at home adj (care or treat* or manage* or intervention*) adj3 (chronic or life limiting or disease or cancer or high symptom burden)).ti,ab. |
| 9 | (home based adj (care or treat* or manage* or intervention*) adj3 (chronic or life limiting or disease or cancer or high symptom burden)).ti,ab. |
| 10 | (community adj (care or treat* or manage* or intervention*) adj3 (chronic or life limiting or disease or cancer or high symptom burden)).ti,ab. |
| 11 | (community based adj (care or treat* or manage* or intervention*) adj3 (chronic or life limiting or disease or cancer or high symptom burden)).ti,ab. |
| 12 | (community centered adj (care or treat* or manage* or intervention*) adj3 (chronic or life limiting or disease or cancer or high symptom burden)).ti,ab. |
| 13 | (community centred adj (care or treat* or manage* or intervention*) adj3 (chronic or life limiting or disease or cancer or high symptom burden)).ti,ab. |
| 14 | 1 or 2 or 3 or 4 or 5 or 6 or 7 or 8 or 9 or 10 or 11 or 12 or 13 |
| 15 | exp Advance Care Planning/ |
| 16 | (advance adj (care or directive* or statement*)).ti,ab. |
| 17 | anticipatory care.ti,ab. |
| 18 | care planning.ti,ab. |
| 19 | (end of life adj2 planning).ti,ab. |
| 20 | living will*.ti,ab. |
| 21 | 15 or 16 or 17 or 18 or 19 or 20 |
| 22 | 14 and 21 |
| 23 | ((patient* or family* or caregiver* or carer*) and (preference* or experience* or reported outcome* or satisfaction or needs or expectation* or goal*)).ti. |
| 24 | ((patient* or family* or caregiver* or carer*) adj2 (preference* or experience* or reported outcome* or satisfaction or needs or expectation* or goal*)).ti,ab. |
| 25 | 23 or 24 |
| 26 | 14 and 25 |
| 27 | ((patient* or family* or caregiver* or carer*) and (navigat* or prepar* or educat* or teach* or inform or informed or informing or skill*)).ti. |
| 28 | ((patient* or family* or caregiver* or carer*) adj2 (navigat* or prepar* or educat* or teach* or inform or informed or informing or skill*)).ti,ab. |
| 29 | 27 or 28 |
| 30 | 14 and 29 |
| 31 | (integrat* adj2 care).ti,ab. |
| 32 | ((interdisciplinary or inter disciplinary or multidisciplinary or multi disciplinary or cross disciplinary or transdisciplinary or trans disciplinary) adj2 team*).ti,ab. |
| 33 | (interprofessional or inter professional or cross professional).ti,ab. |
| 34 | open communication.ti,ab. |
| 35 | (professional* adj2 (partnership* or relationship* or communicat* or collaborat* or cooperat* or coordinat*)).ti,ab. |
| 36 | (task* adj distribution).ti,ab. |
| 37 | 31 or 32 or 33 or 34 or 35 or 36 |
| 38 | 14 and 37 |
| 39 | 22 or 26 or 30 or 38 |

1. **Broad topics for librarian-assisted purposive search**

| Librarian-assisted purposive search topics |  |  |
| --- | --- | --- |
|  | MEDLINE | CINAHL |
| Advance care planning | 57 | 41 |
| Goals/preferences for care | 230 | 159 |
| Patient/family navigation | 66 | 28 |
| Integrated/interprofessional care | 101 | 31 |
| Total, all categories (no dups) | 405 | 250 |
