# Supplementary material for: Engaging family caregivers and health system partners in exploring how multi-level contexts in primary care practices affect case management functions and outcomes of patients and family caregivers at end of life: a realist synthesis
Source: BMC Palliat Care. 2021 Jul 16;20:114. doi: 10.1186/s12904-021-00781-8 (PMC8285870; doi:10.1186/s12904-021-00781-8)
Supplement: Supplementary file 3 — Additional file 3. Six initial theories. [file 12904_2021_781_MOESM3_ESM.docx]

**Additional File 3: Six initial theories**

| **Initial theories** | **Questions to probe for evidence** |
| --- | --- |
| 1: **IF** Primary Care teams have training to facilitate end-of-life conversations with patients/families, **THEN** providers will identify patients nearing end-of-life, involve them in their plan of care, and ensure greater continuity of care in the last year of life | 1: What mechanisms and contexts facilitate Primary Care practices to identify patients at end-of-life (e.g. conversations, tools) and what are the outcomes? |
| 2: **IF** resources/supports are provided to Primary Care teams to conduct end-of-life planning with patients/families, **THEN** plans will be initiated in Primary Care | 2. What mechanisms and contexts facilitate Primary Care teams to plan for end-of-life with patients/families and what are the outcomes? |
| 3: **IF** resources/supports are provided to create/maintain intersectoral data sharing and monitoring of family-centred plans, **THEN** there will be improved intersectoral communication of plans/coordination of care for patients/families | 3: What mechanisms and context facilitate coordination across health, social and community sectors (e.g. data sharing and monitoring) of family-centred plans and what are the outcomes? |
| 4: **IF** resources/supports are provided to adopt a Public Health approach to end-of-life care in the community, **THEN** Primary Care settings would be prompted to partner with critical community resources that would work with citizens and patients/family caregivers for the development of “upstream” end-of-life planning | 4: What mechanisms and context facilitate Primary Care settings to partner with critical community resources (voluntary sector, health and social partners) that would work with citizens and patients/family caregivers for the development of “upstream” end-of-life planning? |
| 5: **IF** resources/supports are provided to adopt a Public Health approach to end-of-life care in the community, **THEN** Primary Care settings would be prompted to include designated positions for community health workers to work with critical community resource partners that work with citizens and patients/family caregivers for the development of “upstream” end-of-life planning | 5: What mechanisms and context facilitate Primary Care settings to include designated positions for community health workers to work with critical community resource partners that would work with citizens and patients/family caregivers for the development of “upstream” end-of-life planning? |
| 6: **IF** resources/supports are provided to adopt a Public Health approach to end-of-life care in the community, **THEN** Primary Care settings would be prompted to work with communities to determine partnerships with critical community supports and assess citizen and patient/family caregivers’ needs for end-of-life planning | 6: What mechanisms and context facilitate Primary Care settings to work with communities to determine partnerships with critical community supports and assess citizen and patient/family caregivers’ needs for end-of-life planning? |
