# Supplementary material for: Engaging family caregivers and health system partners in exploring how multi-level contexts in primary care practices affect case management functions and outcomes of patients and family caregivers at end of life: a realist synthesis
Source: BMC Palliat Care. 2021 Jul 16;20:114. doi: 10.1186/s12904-021-00781-8 (PMC8285870; doi:10.1186/s12904-021-00781-8)
Supplement: Supplementary file 4 — Additional file 4. Tables providing evidence for Theories 1a, 1b, and 2. [file 12904_2021_781_MOESM4_ESM.docx]

Additional File 4: Tables providing evidence for Theories 1a, 1b, and 2

| **Evidence for Program Theory 1a:** | |
| --- | --- |
| **M1_a_-Resources-using tools or frameworks to create opportunities for end-of-life communication** | **Contexts or M1_a_-Reasoning to consider that can affect outcomes** |
| **Tools to identify patients nearing end-of-life:** There will be some unpredictability in the trajectory of progressive life-threatening non- cancerous illnesses, and it is difficult to determine a specific cut-off point to refer patients to palliative care. **Coventry, 2005**  **Tools to assess patient and family needs:** There is evidence that using a tool to assess patients’ and/or families’ needs can present an opportunity to have a conversation about end-of-life and/or create a plan. **Aoun, 2015; Ewing, 2016**  Using an assessment tool can have positive impacts when used as part of a caregiver-led assessment and support process: it made support needs visible, legitimised support for carers and opened up different conversations with carers. **Ewing, 2016**  Patients saw a shared decision-making tool as a vehicle for beginning an important discussion and raising people’s awareness of the topic. They indicated that they expected their providers to ‘step up’ and begin these important discussions rather than ‘dodging’ challenging questions as they had experienced providers doing in the past. **Jones, 2014**  **Tools to communicate with team members:** Using a formal tool to communicate patients' plan of care can improve multidisciplinary care and facilitate discussions regarding patients’ and their family’s or carer’s wishes at the end-of-life. **Gallagher, 2012**  **Using a framework for discussions:** Using a dual framework that focuses on living well while acknowledging the possibility of dying can help clinicians working with seriously ill patients hold both possibilities and encourage those patients to tolerate the possibility of dying and develop a more integrated perspective. It can also create a supportive space for the patient’s experience to emerge and help the patient prepare to talk about the future. **Jacobsen, 2017**  The provision of a framework to enable conversations with patients who wanted to talk about their concerns for the future was viewed to be important. **Seymour, 2010** | **Time:** There may be some resistance from practitioners to using a tool due to the additional time it may take to administer the tool **Aoun, 2015; Jones, 2014**  **Practitioner self-efficacy:** Practitioners perceive their lack of skill in defining the right moment for initiating discussion as a barrier to engaging in advance care planning. **Coventry, 2005**  Practitioners fear tool may identify needs that cannot be met. **Ewing, 2016**  **Prior training:** There may need to be supplemental training on how to communicate with patients **Aoun, 2015**  Training that uses active learner participation and interaction prompted by relevant case discussion increased confidence of practitioners in their ability to use palliative knowledge in the course of their jobs. **Wharton, 2015**  **Creating spaces for difficult conversations:** The clinician needs to be able to create a supportive space for the patient’s experience to emerge and to organize the discussion by providing safety and containment for the discussion. **Jacobsen, 2017**  Opportunities for conversations about patient preferences and goals of care arise when communication about the transition between curative care (hospital narrative) and hospice care (hospice narrative) are shared with patients and families. **Wharton, 2015**  **Normalizing the use of tools in everyday practice:** If a tool was used to improve shared decision making in the context of usual visits or usual care (upstream), patients would view it as a normal part of care, ‘not scary’ rather than a frightening ‘death message (downstream)’. **Jones, 2014**  To facilitate patient screening for palliative care an assessment tool can be a template in the electronic medical record, and part of the usual assessment that takes place for each patient. **Wharton, 2015** |
| **Outcomes:** Starting palliative care as early as possible, at any stage of the illness, along with curative measures would be beneficial for the patients. **Coventry, 2005** | |

| **Evidence for Program Theory 1b** | |
| --- | --- |
| **M1_b_-Resources- learning ways to improve end-of-life communications** | **Contexts or M1_b_-Reasoning to consider that can affect outcomes** |
| **Helpful communication styles:** Communication between health providers and family/friend caregivers that ask care-specific questions, open-ended questions/statements, and personal questions can activate family/friend caregivers to care for patient. Follow up conversations can provide education, reassess the patient/care environment, validate communications, clarify care issues, update/revise care, and make recommendations for future care. **Dingley, 2016**  Having a discussion focused on service provision and availability may be a useful and acceptable step toward a discussion on their end-of-life wishes. **Holdsworth, 2011**  The findings show that “social support was identified as a crucial resource to support self-care behaviour” by patients. **Johnston, 2009**  Building rapport, and having communication with patients about their conditions, and what may come up prepare patients for the future. All of this, beside the interprofessional communication, provide “a community of professionals” that help patients move through the illness trajectory with comfort and trust. There are three different types of illness journeys: Isolated journey, rescued journey, and comforted journey. These findings indicate embedding palliative care into patients care as early as possible. **Wittenberg-Lyles, 2011**  Candid communication about treatment options in line with patient and family goals leads to fewer aggressive life-sustaining medical interventions near death, as well as better overall bereavement experiences for the family. **Wittenberg-Lyles, 2011**  Preparing patients for end-of-life allowed them to remain in control and be prepared for their impending death. Patients also received information on finding comfort and reaching closure. **Johnston, 2009**  **Triggers for conversations:** Admission or discharge of patients from hospital three can trigger discussions between GPs and patients. **Le, 2017**  Training/education in conversation starters improved context for having end-of-life conversations **Blackford, 2013**  There is a need for training of family physicians and others in primary care and the need for training on how to start conversations was noted. **Howard, 2018** | **Time/Resources:** Need time to have meaningful conversations (Dingley, 2016)  Discussing end-of-life preferences was not always a primary goal of care planning. **Holdsworth, 2011**  The need for more effective communication has implications on the time healthcare practitioners spend with patients and their families and practice routines that provide time. **Ventura, 2014**  Lack of resources is a barrier to supporting family/friend caregivers. **Thomas, 2010**  General practitioners who anticipate potential end-of-life events and allow for the creation of space for discussion where the patient may verbalize his or her lived experience. Discussing and anticipating potential events allows GPs to collaboratively devise “end-of-life projects” with their patients **OudeEngberink, 2017**  **Relationships and trust with patients:** Building relationships with patients and caregivers made it easier to talk about dying. **Holdsworth, 2011**  The family/friend caregiver felt left out and had feelings of powerlessness when they did not manage to establish a relationship with the healthcare professionals. To achieve a genuine meeting between the care provider and the family/friend caregiver, the caregiver has to have an opportunity to create a dialogue and to feel that someone is listening to him/her at that very moment. **Linderholm, 2010**  Family members reported tremendous appreciation and trust they felt in reliance on practitioners who had cultivated meaningful, long-term caring relationships with the older adult. **Kramer, 2013**  Conversations rely on truth telling, which means being honest about what practitioners do not know, as well as the inherent uncertainty that pervades medicine. **Thomas, 2010**  When the health professionals did not notice the family caregiver’s feelings of insecurity and inexperience, the outcome was a feeling of powerlessness.  **Linderholm, 2010**  **Disciplinary backgrounds may affect conversation skills:** Social workers were perceived to help families by providing information and emotional support and reducing family burden. **Kramer, 2013**  Offering palliative care was perceived by General Practitioners as a moral obligation. Discussing and anticipating potential events allows General Practitioners to collaboratively devise “end-of-life projects” with their patients and share care amongst a multi-disciplinary team. **OudeEngberink, 2017**  General practitioners may not have the confidence to provide palliative care because of patient complexity, inadequate training and insufficient resources. Other barriers included poor communication from specialists and treating teams. **Le, 2017**  The most common reasons nurses articulated for the absence of ACP were a conviction that these discussions were the work of the social worker or counselor and the feeling that either 'the training that they had wasn't enough, or they just didn't feel confident enough to bring up some of those subjects. **Blackford, 2013**  **Poor communication skills affect patients:** Findings show poor communication, mixed messages, unrealistic information resulted in patients’ unrealistic expectations (mentioned by healthcare providers) and feeling of fear and uncertainty about the palliative care. **Gardiner, 2015**  Communication issues health care providers and family, friends, or health-care delivery systems negatively affect patient care. **Kelley, 2013**  **Patient or Family/Friend Caregivers preferences:** Having more knowledge about what to expect of the dying process, knowing their relative’s wishes, and understanding the role of hospice and palliative care could improve the caregivers experience of events leading up to death. The patient’s understanding of their prognosis was felt to be a precondition for an end-of-life discussion by health professionals. **Holdsworth, 2011**  There is a patient willingness even in the advanced stage of cancer to assume responsibility for their own treatment. **Johnston, 2009**  Family/friend caregivers wish to have information, conversations and support to enable them to make choices. **Thomas, 2010** |
| **Outcomes**  **Communication has an effect on patient and family/friend caregiver outcomes:** Late recognition of palliative care need and referrals at a late stage can have a negative impact on patients, and on their relatives during bereavement **Gardiner, 2015**  Family/friend caregivers identified communication and teamwork Issues as important barriers. **Kelley, 2013**  The most frequently reported unmet need of palliative home care patients and their family/friend caregivers was effective communication with healthcare professionals, the lack of which negatively impacted on the care received. **Ventura, 2014** | |

| **Evidence for Program Theory 2** | |
| --- | --- |
| **M2-Resources - Facilitators for Advance Care Planning** | **Contexts or M2-Reasoning to consider that can affect outcomes** |
| **Using tools in Advance Care Planning:** An Advance Care Planning-Service Evaluation Tool implemented in community palliative care that identified advance care planning progress over time was used by practitioners either as a peer-assessment or self-assessment tool to help track implementation progress as well as plan further change strategies. The tool enabled monitoring, evaluation and planning of quality improvement for advance care planning. **Blackford, 2012**  **Group visits:** Participation in an advance care planning group visit intervention for older adults significantly increased advance care planning documentation of surrogate decision makers and goals for future medical care among older adults in primary care clinics. advance care planning group visit can be implemented at the practice level*.* **Lum, 2017**  **Using opportunities during patient conversations:** The provision of a framework to enable conversations with patients who wanted to talk about their concerns for the future. Being aware when patients introduce issues about the end-of-life that it can be a hook to hang the next piece of conversation on while following the lead of the patient. Adapting the pace of the conversation to the patient’s degree of comfort with what otherwise may be dangerous territory. **Seymour, 2010**  **Effective education program for practitioners:** The following are suggestions for what should be included in education programmes: design of realistic scenarios; design of a flow chart; practical advice about communication and documentation; insights into the need for clinical supervision for advance care planning practice. **Sanders, 2008**  **Positive effects of Advance Care Planning:** In-home end-of-life care programs with patient centric care planning demonstrated a significant cost reduction. For instance, programs that included linkage with other community providers or primary care tended to have positive outcomes in a high proportion of studies. **Bainbridge, 2016**  Advance care planning is associated with a lower rate of in-hospital death. There appears to be utility in each aspect of advance care planning (i.e. completing an advance directive, assigning a durable power of attorney and having an advance care planning discussion with next of kin). **Bischoff, 2013** | **Time/Resources*:*** Introducing new concepts such advance care planning one size doesn’t fit all. It is important to be aware of in which context we are going to talk about planning for death, and also, building relationship and providing adequate and correct information/expectation are necessary. **Sanders, 2008**  Inadequate resourcing was identified as a key barrier to the implementation of advance care planning. There needed to be adequate services and resources in place to engage with advance care planning, to support any choices that patients might record for their future care towards the end-of-life and provide support to family carers. **Seymour, 2010**  Timing can be a barrier to advance care planning and also how to effectively work in teams. **Seymour, 2010**  **Creating space for difficult conversations:** Early recognition of the palliative transition point can be key to ensuring end of life issues are addressed. **DeVleminck, 2013**  **Practitioner embracing facilitator role:** Referral from primary care providers to advance care planning group visit may be a key implementation strategy; some participants suggested that it could be helpful to discuss one’s wishes with family or friends ‘‘one at a time’’ or to include discussions with an ‘‘outside person that’s neutral’’ such as a social worker or physician. **Lum, 2017**  Practitioners can act as a facilitator to begin to engage patients in the advance care planning process. **Sudore, 2017**  **Relationships and trust with patients:** It is important to be aware of what context we are going to talk about planning for death, and also, building relationship and providing adequate and correct information/expectation are necessary. **Sanders, 2008**  Fear of patients not being ready for advance care planning, depriving a patient of hope, and subsequently damaging the practitioner – patient relationships were cited as factors that keep practitioners from engaging in the process of Advance Care Planning. **DeVleminck, 2013**  The objective to reduce costs might work against humane values, dignity and choices regarding end-of-life care. Of course, the issues of choice and participation are also strong themes of current health policy discourse around chronic disease management. **Sanders, 2008**  **Patient or Family/Friend Caregivers awareness:** Lack of public awareness about advance care planning and difficulties in talking about death is a barrier to discussing advance care planning. **Seymour, 2010**  End of life care is surrounded by a ‘curative’ culture which forecloses on the possibility of preparation for death and poses a barrier to planning supportive services for dying patients and their families. **Seymour, 2010**  **Disciplinary backgrounds may affect readiness to do advance care planning:** General practitioners are often reluctant to consider and discuss specific decisions relating to advance care planning with patients or their representatives. It was felt that this reluctance arose from discomfort among General practitioners about raising any advance care planning issues with patients, for fear of raising issues about the end-of-life ‘too soon’. **Seymour, 2010** |
| **Outcomes**  Reduction of costs associated with in-home end-of-life care programs and patient centric end-of-life care planning that included linking primary care with community resources. **Bainbridge, 2016**  Advance care planning was found to be associated with a lower rate of in-hospital deaths. **Bischoff, 2013** | |
